# Rationalizing general limitations in assessing and comparing methods for compound potency prediction

**DOI:** 10.1038/s41598-023-45086-3

**Published:** 2023-10-19

**Authors:** Tiago Janela, Jürgen Bajorath

**Affiliations:** B-IT, LIMES Program Unit Chemical Biology and Medicinal Chemistry, Department of Life Science Informatics and Data Science, Rheinische Friedrich-Wilhelms-Universität, Friedrich-Hirzebruch-Allee 5/6, 53115 Bonn, Germany

**Keywords:** Cheminformatics, Machine learning

## Abstract

Compound potency predictions play a major role in computational drug discovery. Predictive methods are typically evaluated and compared in benchmark calculations that are widely applied. Previous studies have revealed intrinsic limitations of potency prediction benchmarks including very similar performance of increasingly complex machine learning methods and simple controls and narrow error margins separating machine learning from randomized predictions. However, origins of these limitations are currently unknown. We have carried out an in-depth analysis of potential reasons leading to artificial outcomes of potency predictions using different methods. Potency predictions on activity classes typically used in benchmark settings were found to be determined by compounds with intermediate potency close to median values of the compound data sets. The potency of these compounds was consistently predicted with high accuracy, without the need for learning, which dominated the results of benchmark calculations, regardless of the activity classes used. Taken together, our findings provide a clear rationale for general limitations of compound potency benchmark predictions and a basis for the design of alternative test systems for methodological comparisons.

## Introduction

In computer-aided drug discovery, the prediction of compounds that are active against given targets and the prediction of compound potency are central tasks^1, 2^. For the quantitative prediction of compound potency, methods of greatly varying complexity have been introduced, ranging from linear regression techniques to deep machine learning^3–8^. For modeling of non-linear structure-activity relationships and potency prediction, machine learning has generally become the prevalent approach, for which a variety of algorithms are available^1, 5^. Despite the increasing popularity of deep neural networks^7, 8^, mainstay approaches such as random forest regression (RFR)^9^ or support vector regression (SVR)^10^ continue to be widely used.

Computational methods for qualitative compound activity or quantitative potency predictions must generally be evaluated in benchmark settings using known active compounds. For activity prediction, classification models are often trained to separate sets of compounds that are active against different targets, termed activity classes, from randomly assembled compounds. Hence, activity classes represent target-based compound data sets (target sets). For potency prediction, regression models are derived for individual activity classes to predict potency values of test sets extracted from these classes. Care should be taken to limit model derivation and evaluation to compounds for which well-defined potency measurements of the same type are available that can be directly compared. Hence, data curation plays an important role.

Although benchmarking is not a reliable indicator for the success or failure of alternative approaches in practical applications, it represents an essential first step in performance evaluation and comparison of different methods. However, for compound potency prediction, principal limitations of benchmark calculations were recently uncovered^11^. Specifically, it was shown that (i) different machine learning methods including deep neural networks produced very similar potency predictions on different activity classes; (ii) simple k-nearest neighbor (kNN) assignments, carried out as a control, correctly predicted potency values within an order of magnitude, comparable to increasingly complex machine learning methods; (iii) random predictions often also reproduced experimental potency values within an order of magnitude; (iv) prediction errors of all methods fell into a small interval^11^. Overall, SVR predictions were slightly more accurate than those obtained with other methods including deep neural networks, but observed differences were only marginal^11^. Hence, typical benchmark calculations yielded predictions of comparable accuracy using distinct methods of varying computational complexity as well as random predictions. It follows that standard benchmark calculations are not suitable for assessing the predictive performance of machine learning methods in a meaningful way. The generality of these unexpected findings was further investigated by systematic potency predictions using machine learning methods and controls on 367 activity classes covering all pharmaceutical target classes including, among others, diverse enzymes, different types of receptors, and ion channels, for which qualifying potency measurements were available, which yielded very similar results^12^. Taken together, these findings suggested that the intrinsic limitations of benchmark potency predictions might be a consequence of the composition of activity classes originating from medicinal chemistry sources and their potency value distributions. Therefore, activity classes were modified in different ways including removal of nearest neighbors, partitioning of compounds into training and test sets based on analogue series (thereby avoiding “data leakage”, that is, the use of analogous compounds for training and testing), and balancing of compound numbers across different potency levels^12^. Then benchmark calculations were repeated with modified activity classes. However, the predictions were surprisingly stable and largely insensitive to these data set modifications, leading to only small increases in error margins that were very similar for different methods^12^. Thus, reasons for the very similar performance of different methods and simple controls in compound potency predictions remained elusive.

Therefore, we have further investigated potential reasons for the limitations of compound potency predictions. Since the predictions were essentially insensitive to structural modifications of activity classes, we have conducted an in-depth analysis of the influence of potency value distributions and potency sub-ranges in activity classes on compound potency predictions using different approaches, as reported herein.

## Methods

### Compounds and activity data

**Figure 1:**
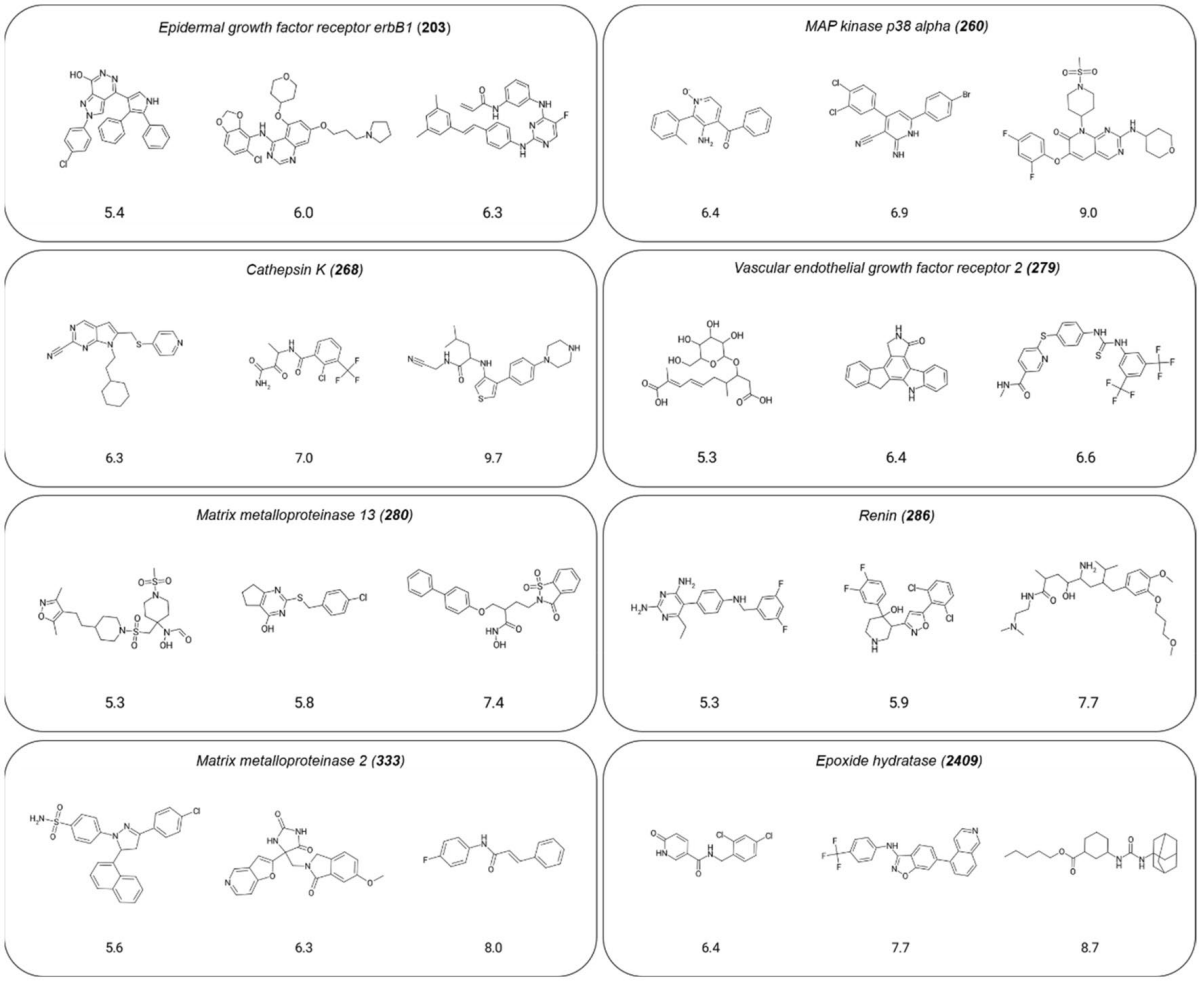
Activity classes. For each of the eight activity classes (target sets), the target name and ChEMBL target ID (in parentheses) are provided and exemplary structurally diverse compounds are shown. For each compound, the pIC_50_ value is reported.

From ChEMBL (version 30)^13^, compounds with reported direct interactions (target relationship type: “D”) with human targets at the highest confidence level (target confidence score: 9), a molecular mass of at most 1000 Da, and available numeric IC_50_ values (recorded as negative decadic logarithmic pIC_50_ values) in the range of 5–11 were extracted. Compounds with measurements flagged as “potential transcription error” or “potential author error’’ were discarded as well as compounds with assay interference potential detected using available filters^14–16^. We searched for activity classes for which at least 75 compounds falling into each of the three potency pIC_50_ sub-ranges 5–6.9, 7–8.9, and 9–11 were available, leading to the identification of eight classes comprising a total of 9301 compounds. In the following, for simplicity, these sub-ranges are referred to as 5–7, 7–9, and 9–11. Figure 1 shows exemplary compounds for each class and specifies the target names.

### Training and test sets

For each activity class, training and test sets for 10 independent prediction trials were obtained by random compound partitioning into 50% training and 50% test data. Hence these training and test sets were not balanced across the three potency sub-ranges. Supplementary Table S1 reports the proportions of compounds falling into each potency sub-range for all activity classes. For the three activity classes with the largest number of compounds in the potency sub-range 9–11 (highly potent compounds), nine training sets of increasing size were generated (for 10 independent trials) by uniformly sampling compounds for each potency sub-range. Smallest training sets consisted of only six compounds (two from each potency sub-range), followed by training sets with 12 compounds (four per potency sub-range), 18, 30, 48, 78, 126, 204, and 330 compounds. After building the largest training set (330 compounds), the remaining compounds, were used to build the test set with balanced potency sub-ranges (with respect to sub-range 9–11, containing the smallest number of compounds per sub-range for the three activity classes).

For comparison, corresponding predictions were also carried out for imbalanced training sets of increasing size and imbalanced test sets.

### Molecular representation

For machine learning, compounds were represented using the folded 2048-bit version of the extended connectivity fingerprint with bond diameter 4 (ECFP4)^17^ generated with RDKit^18^.

### Machine learning models

Given that potency prediction results were very similar using methods of different complexity and simple controls^11^, machine learning models were built using SVR, the overall preferred approach, and RFR for comparison.

#### Hyperparameter optimization

For hyperparameter optimization, a grid search with 3-split cross-validation was performed using *scikit-learn*^19^ based on training data. Therefore, training sets were divided into 50% training and 50% validation data. For balanced training sets, the splits were stratified by potency range.

#### Support vector regression

SVR is a variant of the support vector machine algorithm for supervised learning that derives a hyperplane based on training instances to reduce the error between observed and predicted values. A kernel function is used to project samples from the original dimension into a higher-dimensional feature space^10, 20^. For SVR, the cost parameter C was optimized with the values of 1, 10, 100, and 1000. Models with the Tanimoto kernel^21^ were built using *scikit-learn*.

#### Random forest regression

RFR is a machine learning method employing an ensemble of decision trees. Each tree model was built by randomly sampling a subset of training compound using bootstrapping^9, 22^. Numerical values were predicted as the average value of all individual trees. For RFR, the number of trees (50, 100, 200), minimum number of samples per split (2, 3, 5, 10), minimum sample per leaf (1, 2, 5, 10), and maximal number of features for achieving the best split (sqrt, log2) were optimized.

### Controls

#### Nearest neighbor calculations

k-NN is a regression technique that selects for each test instance the k nearest neighbors from the training set and assigns the potency value of the most similar training compound to the test instance (1-NN) or averages the potency values for the k (> 1) most similar training compounds^23^. For comparing test and training set compounds, Tanimoto similarity^24^ was calculated based on ECFP4. 1-NN and 3-NN calculations were carried out with *scikit-learn.*

#### Median regression

Median regression (MR), the simplest possible control, assigns the median potency value of the training set to each test compound as the predicted value.

### Performance metrics

Prediction accuracy was evaluated using the mean absolute error (MAE), root mean squared error (RMSE), and squared Pearson correlation coefficient (r^2^). Training of machine learning models was guided by MAE values or, as a control, R^2^ (coefficient of determination).

1 $$MAE\left( {y,\hat{y}} \right) = \frac{1}{n}\mathop \sum \limits_{i = 1}^{n} \left| {y_{i} - \hat{y}_{i} } \right|$$

2 $$RMSE\left( {y, \hat{y}} \right) = \sqrt {\mathop \sum \limits_{i = 1}^{n} \frac{{\left( {y_{i} - \hat{y}_{i} } \right)^{2} }}{n}}$$

3 $$r^{2}= \left( {\frac{{\sum \left( {x - m_{x} } \right)\left( {y - m_{y} } \right)}}{{\sqrt {\sum \left( {x - m_{x} } \right)^{2} \sum \left( {y - m_{y} } \right)^{2} } }}} \right)^{2}$$

For MAE and RMSE, *n* is the number of compounds, and $y$ and $\hat{y}$ are the experimental and predicted potency values, respectively. For r^2^, $m_{x}$ is the mean of vector $x$ and $m_{y}$ the mean of vector $y$.

### Statistical significance testing

The Wilcoxon signed-rank test^25^ was used to assess the statistical significance of observed differences between MAE, RMSE and r^2^ value distributions. The p-value (*p* < α) was compared to an alpha value of 0.005 with Bonferroni correction (n = 10).

## Results

### Compound potency value distributions

**Figure 2:**
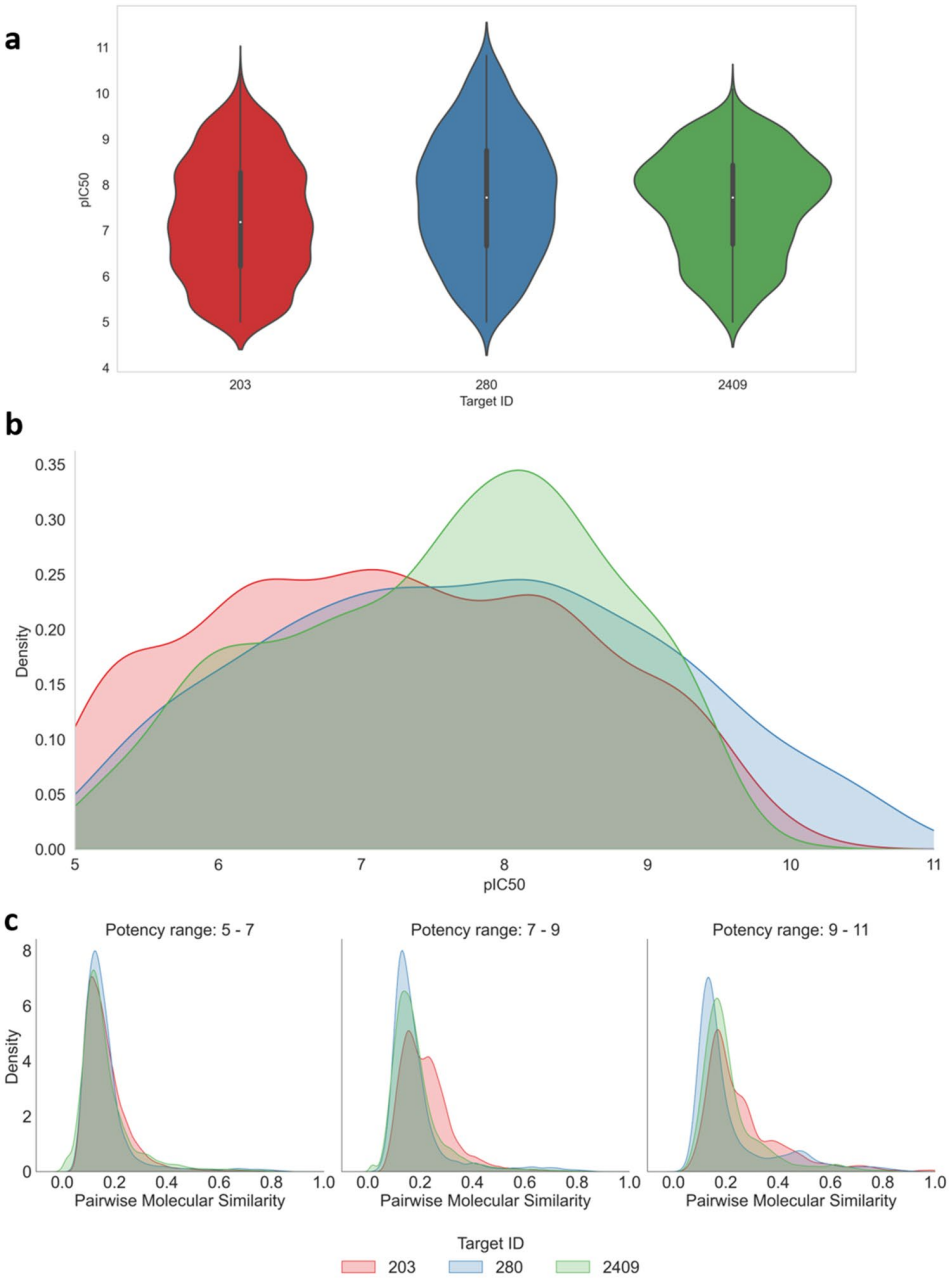
Potency value and pairwise molecular similarity distributions. For the three activity classes with the largest numbers of compounds in potency sub-range 9–11, (**a**) violin plots report the potency value distributions across the three potency sub-ranges (5–7, 7–9, 9–11). In a violin plot, a value distribution is represented by its maximum value (upper thin line), upper quartile (upper thick line), median value (white dot), lower quartile (lower thick line) and minimum value (lower thin line). On each side of the vertical line, a density plot is shown. In (**b**), density plots obtained by kernel density estimation compare the potency distributions across the entire potency range. In (**c**), density plots report the distributions of pairwise Tanimoto similarity values for compounds populating the three potency sub-ranges.

We first determined the potency value distributions of the activity classes, as shown in Fig. 2a and Fig. 2b for the three classes with largest numbers of compounds in potency (pIC_50_) sub-range 9–11 and Supplementary Fig. S1a and Fig. S1b for all classes. The center of the entire potency range 5–11 corresponding to compounds with intermediate potency contained the majority of compounds in all classes. In each case, the median potency of all classes fell into the pIC_50_ interval 7–8. However, there were clear activity class-dependent differences in potency value distributions, with different peaks in the distributions.

Pairwise similarity was then separately calculated for all compounds falling into each of the potency sub-ranges 5–7, 7–9, and 9–11. Figure 2c and Supplementary Fig. S1c show that the resulting similarity value distributions were comparable for all activity classes and also comparable for each class across the different potency sub-ranges. As expected, some activity classes were structurally more homogeneous than others in individual sub-ranges (such as class 203 in Fig. 2), but large differences in compound similarity value distributions across different potency sub-ranges were not observed. Hence, there was no apparent relationship between intra-class compound similarity and differences in potency value distributions between the activity classes.

### Compound potency predictions

**Figure 3:**
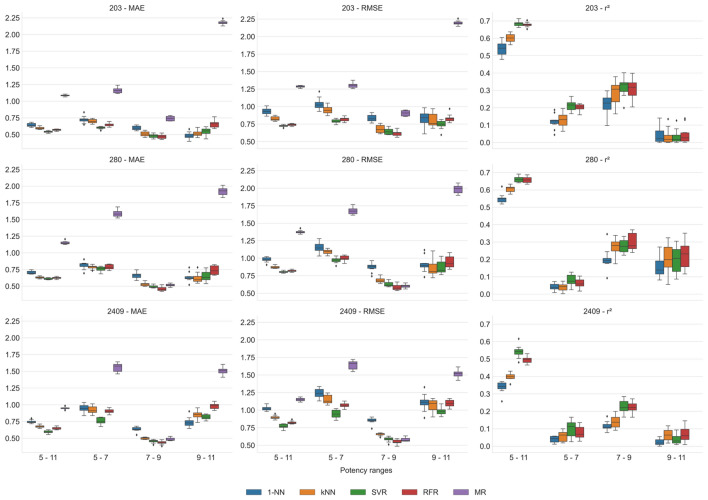
Prediction accuracy. Boxplots report the distribution of MAE (left), RMSE (middle), and r^2^ values (right) for potency predictions over 10 independent trials with constantly sized (imbalanced) training sets using 1-NN, 3-NN, SVR, RFR, and MR for three activity classes. In each case, predictions are reported for the entire potency range (5–11) and test compounds with experimental potency falling into the three sub-ranges. In boxplots, the upper and lower whiskers indicate maximum and minimum values, the boundaries of the box represent the upper and lower quartiles, values classified as statistical outliers are shown as diamonds, and the median value is indicated by a horizontal line.

For the eight activity classes, potency predictions were carried out using the SVR, RFR, 1-NN, 3-NN, and MR approaches. Prediction accuracy was assessed on the basis of MAE, RMSE, and r^2^ calculations. Figure 3 shows the results for the three activity classes with the largest numbers of highly potent compounds and Supplementary Fig. S2 compares the results for all activity classes based on MAE (Fig. S2a), RMSE (Fig. S2b), and r^2^ values (Fig. S2c). Consistent with earlier observations^11, 12^, the performance of all methods across the entire potency range was comparable for all activity classes and varying training and test set ratios. The predictions were stable, as indicated by very narrow error distributions over independent trials, and reached reasonable accuracy, with MAE and RMSE values generally smaller than 0.8 and 1.0, respectively (except for MR, as further discussed below). Hence, the different methods generally predicted potency values well within an order of magnitude (tenfold). Lowest prediction errors detected were ~ 0.4 and ~ 0.5 for MAE and RMSE, respectively. SVR predictions were overall slightly more accurate than RFR and 1-/3-NN calculations. As a control, the machine learning models were also retrained using R^2^ as a cost function and the predictions using these models were assessed based on MAE values. As shown in Supplementary Fig. S2a and Fig. S2d, the results obtained for alternatively trained models using MAE or R^2^ for alternatively trained models were nearly identical.

Importantly, while the majority of differences between MAE and RMSE value distributions for all pairwise comparisons of methods were statistically significant, as shown in Supplementary Fig. S3a and S3b, respectively, differences in mean prediction errors of all methods were confined to ~ 0.1 units and thus essentially negligible. These results were stable for varying training and test set ratios. In addition, Supplementary Fig. S3c shows that most differences between r^2^ values for potency ranges 5–7 and 9–11 were not statistically significant, hence indicating the presence of strong correlation.

The predictions were then separately compared for all test compounds falling into each of the three potency sub-ranges, as also shown in Fig. 3 and Supplementary Fig. S2, which provided a more differentiated view of the results. For weakly potent (sub-range 5–7) and highly potent (9–11) compounds, prediction errors increased by up to ~ 0.2 units for SVR, RFR, and 1-/3-NN. For MR, MAE/RMSE values up to 2.0 were observed because the median potency value of all activity classes fell into the pIC_50_ range of 7–8 (see above). By contrast, for test compounds in potency sub-range 7–9, prediction errors further decreased for all methods by ~ 0.1 units compared to the global accuracy (potency range 5–11) and was closely matched by MR. The comparison in Fig. 3 indicated that the global prediction accuracy of all methods was essentially determined by the similarly low prediction error observed for all methods in intermediate potency sub-range 7–9 where all compound potency values tended to be close to the median.

Furthermore, as shown in Fig. 3, calculation of r^2^ for predicted and experimental potency values revealed positive correlation across the entire potency range for SVR, RFR and 1-/3-NN predictions (as anticipated, given the low prediction errors and large sample sizes). For the three potency sub-ranges, correlation was significantly lower, which was at least in part attributable to the small sample sizes for the low and high potency sub-ranges. Largest correlation was observed for the mid sub-range (7–9), consistent with the low prediction errors in this range. Importantly, r^2^ calculations did not lead to a larger separation between the performance of different models. Thus, correlation analysis mirrored the observed prediction characteristics discussed above.

### Potency value sub-range dependence of predictions

**Figure 4:**
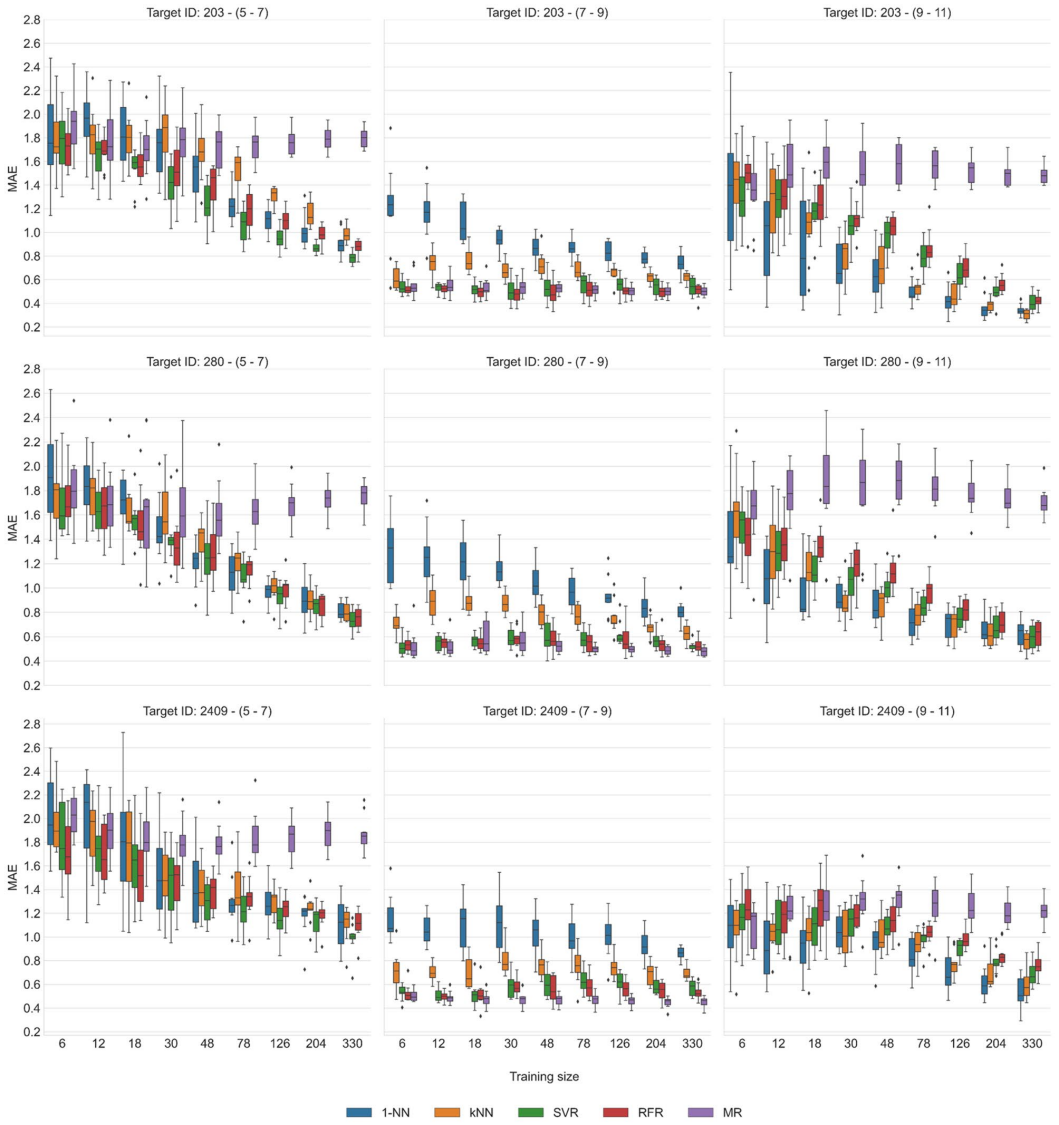
Prediction accuracy for training sets of increasing size. Boxplots report the distribution of MAE values for potency predictions over 10 independent trials with potency sub-ranged balanced training sets of increasing size using 1-NN, 3-NN, SVR, RFR, and MR for three activity classes. The predictions were separately carried out for each potency sub-range.

To further investigate the apparent dependence of the predictions on the compound potency sub-ranges of the activity classes, we generated training sets with balanced sub-range populations of increasing size for the three activity classes for which sufficient numbers of highly potent compounds (see Methods) were available and repeated the predictions for each sub-range. Size variation of training sets was introduced to examine data requirements for the predictions and learning characteristics of the methods. Figure 4 shows the results of sub-range based potency predictions.

For weakly potent (sub-range 5–7) and highly potent (9–11) compounds, smallest training sets of 6–18 compounds produced median MAE values of ~ 2.0 (corresponding to ~ 100-fold potency prediction errors) for all methods and yielded broad MAE value distributions, indicating unstable predictions that were often comparable MR. As expected, very small training sets were insufficient for machine learning and a median MAE of ~ 2.0 essentially represented the upper limit of prediction errors observed under these conditions. When the size of training sets further increased, the predictions for weakly and highly potent compounds became more stable and accurate for SVR, RFR, and 1-/3-NN, as indicated by increasing separation from the MR values, and approached the accuracy level observed in the global predictions (Fig. 3). Hence, for weakly and highly potent test compounds, prediction accuracy clearly increased with the size of training sets with balanced potency sub-ranges, as expected. Notably, the relative performance of the different methods remained comparable as training set sizes and prediction accuracy increased.

By contrast, distinct prediction characteristics were observed for test compounds in potency sub-range 7–9. Here, the prediction errors were constantly small, independent of training set size, and the accuracy achieved by SVR and RFR was very close to the median potency values of the training sets. Thus, in this case, essentially no learning was required and prediction accuracy was constantly high for MR across all training sets. Whereas 1-/3-NN closely matched SVR/RFR predictions for highly and weakly potent compounds, NN calculations mostly yielded larger errors in the intermediate potency sub-range, especially 1-NN. However, most of these NN calculation errors were comparable to the best predictions achieved with all methods for highly and weakly potent test compounds based on largest training sets. Moreover, SVR, RFR, and MR predictions in the potency sub-range 7–9 were consistently the by far most accurate predictions that were obtained. As an additional control, we also repeated the potency sub-range predictions with imbalanced training sets of increasing size, as shown in Supplementary Fig. S4, yielding the same trends.

The analysis of the potency sub-range dependence of the predictions clearly demonstrated that they were largely determined by compounds falling into the intermediate potency range. Here, predictions for machine learning models were consistently most accurate. However, there was essentially no learning required because the predictions were independent of training set sizes and closely matched the median potency values of the training sets. Hence, in the intermediate potency sub-range, predictions yielded artificially low errors, due to narrow local potency value distributions around the median. In original activity classes, the majority of compounds fell into the intermediate potency range, which strongly dominated global potency predictions.

## Conclusion

Compound potency predictions play an important role in computer-aided drug discovery. Benchmark calculations are essential and widely applied for an initial assessment and comparison of predictive methods, prior to practical applications. However, previous studies have revealed general limitations of benchmark evaluation of potency prediction methods. Increasingly complex machine learning methods and simple control calculations displayed similar performance in test calculations on many different activity classes, and even random predictions were only separated from machine learning results by small error margins. As a consequence, benchmarking can currently not reliably assess the predictive performance and relative differences between alternative methods; a conundrum for method development and evaluation. Since the performance of distinct potency prediction approaches was comparable for many different activity classes (as also shown herein), these observations must in principle be attributable to intrinsic features of activity classes such as structural composition or potency value distributions, as we have reasoned. However, origins of apparent artifacts in benchmarking potency prediction methods have remained unknown so far, presenting a substantial problem for the field. Therefore, we have designed test calculations to directly investigate the influence of potency value distributions and sub-range effects on compound potency predictions. Although potency value distributions of activity classes differed, predictions were largely determined by very low errors consistently detected in the intermediate potency (pIC_50_) sub-range 7–9 into which median potency values of different activity classes fell. These prediction characteristics fundamentally differed from those observed for weakly and highly potent compounds. Machine learning predictions in the intermediate potency sub-range consistently and closely matched median potency values of training sets even under learning conditions where predictions of weakly and highly potent compounds essentially failed. The dominance of very low errors in the intermediate potency sub-range led to closely comparable results of different approaches in global potency predictions and provided a clear rationale for the artificial outcome of benchmark calculations including the low error margins hindering methodological comparisons. Taken together, the results of our analysis explain in detail why conventional benchmark settings do not provide a realistic assessment of compound potency prediction methods and provide a basis for future work investigating alternative approaches for more reliable methodological comparisons.

### Supplementary Information

Supplementary Information.

## Data Availability

Calculations were carried out using publicly available software and compound data. Code used for this analysis and the curated activity classes are freely available via the following links: https://github.com/TiagoJanela/Limitations-compound-potency-predictions and https://zenodo.org/badge/latestdoi/663107456.
