# Supplementary material for: Rationalizing general limitations in assessing and comparing methods for compound potency prediction
Source: Sci Rep. 2023 Oct 19;13:17816. doi: 10.1038/s41598-023-45086-3 (PMC10587074; doi:10.1038/s41598-023-45086-3)
Supplement: Supplementary file 1 — Supplementary Information. [file 41598_2023_45086_MOESM1_ESM.docx]

Supplementary Figures

**Rationalizing general limitations in assessing and comparing methods for compound potency prediction**

Tiago Janela and Jürgen Bajorath*

Department of Life Science Informatics and Data Science, B-IT, LIMES Program Unit Chemical Biology and Medicinal Chemistry, Rheinische Friedrich-Wilhelms-Universität, Friedrich-Hirzebruch-Allee 5/6, D-53115 Bonn, Germany.

**Figure legends**

Supplementary Figure S1. Potency value and pairwise molecular similarity distributions. For all eight activity classes, (a) violin plots report the potency value distributions across the three potency sub-ranges (5-7, 7-9, 9-11). In (b), density plots obtained by kernel density estimation compare the potency distributions across the entire potency range. In (c), density plots report the distributions of pairwise Tanimoto similarity values for compounds populating the three potency sub-ranges.

Supplementary Figure S2. Prediction accuracy. For all activity classes, boxplots report the distribution of (a) MAE, (b) RMSE, and (c) r^2^ values for potency predictions over 10 independent trials with constantly sized (imbalanced) training sets using 1-NN, 3-NN, SVR, RFR, and MR for three activity classes. In each case, predictions are reported for the entire potency range (5-11) and test compounds with experimental potency falling into the three sub-ranges. In (d), the distributions of corresponding MAE values are reported for models trained using R^2^ (coefficient of determination) as a cost function (instead of MAE).

Supplementary Figure S3. Statistical significance assessment. Wilcoxon signed-rank tests based on (a) MAE, (b) RMSE, and (c) r^2^ values were carried out to determine the statistical significance of performance differences between SVR, RFR, 1-NN, and 3-NN predictions and MR, as reported in in Fig. 3 and Fig. S2. Red cells indicate p-values above α = 0.005 (no statistical significance) and green cells p-values below α = 0.005 (statistical significance).

Supplementary Figure S4. Prediction accuracy for training sets of increasing size. Boxplots report the distribution of (a) MAE and (b) r^2^ values for potency predictions over 10 independent trials with imbalanced training sets of increasing size using 1-NN, 3-NN, SVR, RFR, and MR for three activity classes. The predictions were separately carried out for each potency sub-range.

**Fig. S1**


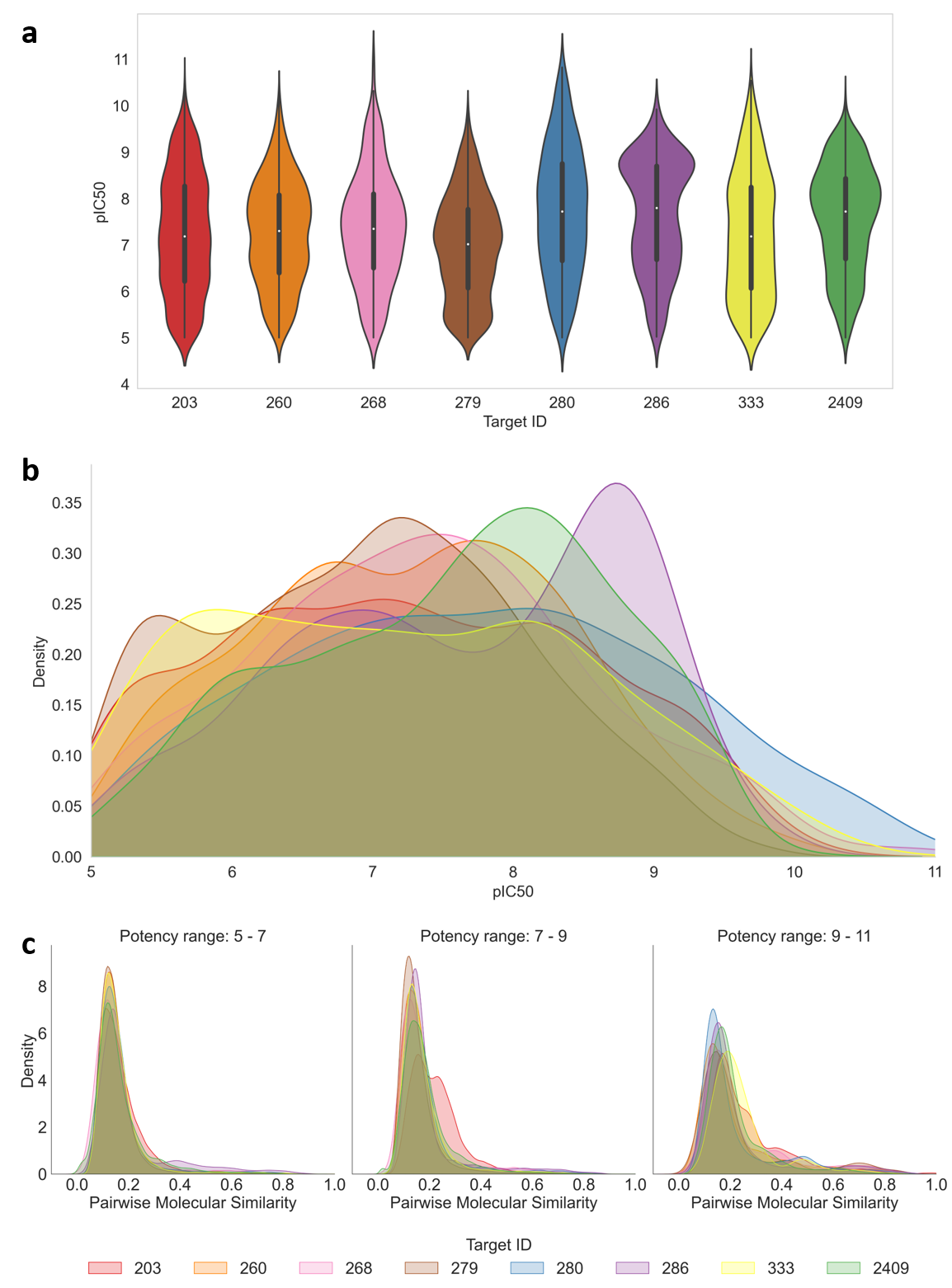


**Fig. S2a**


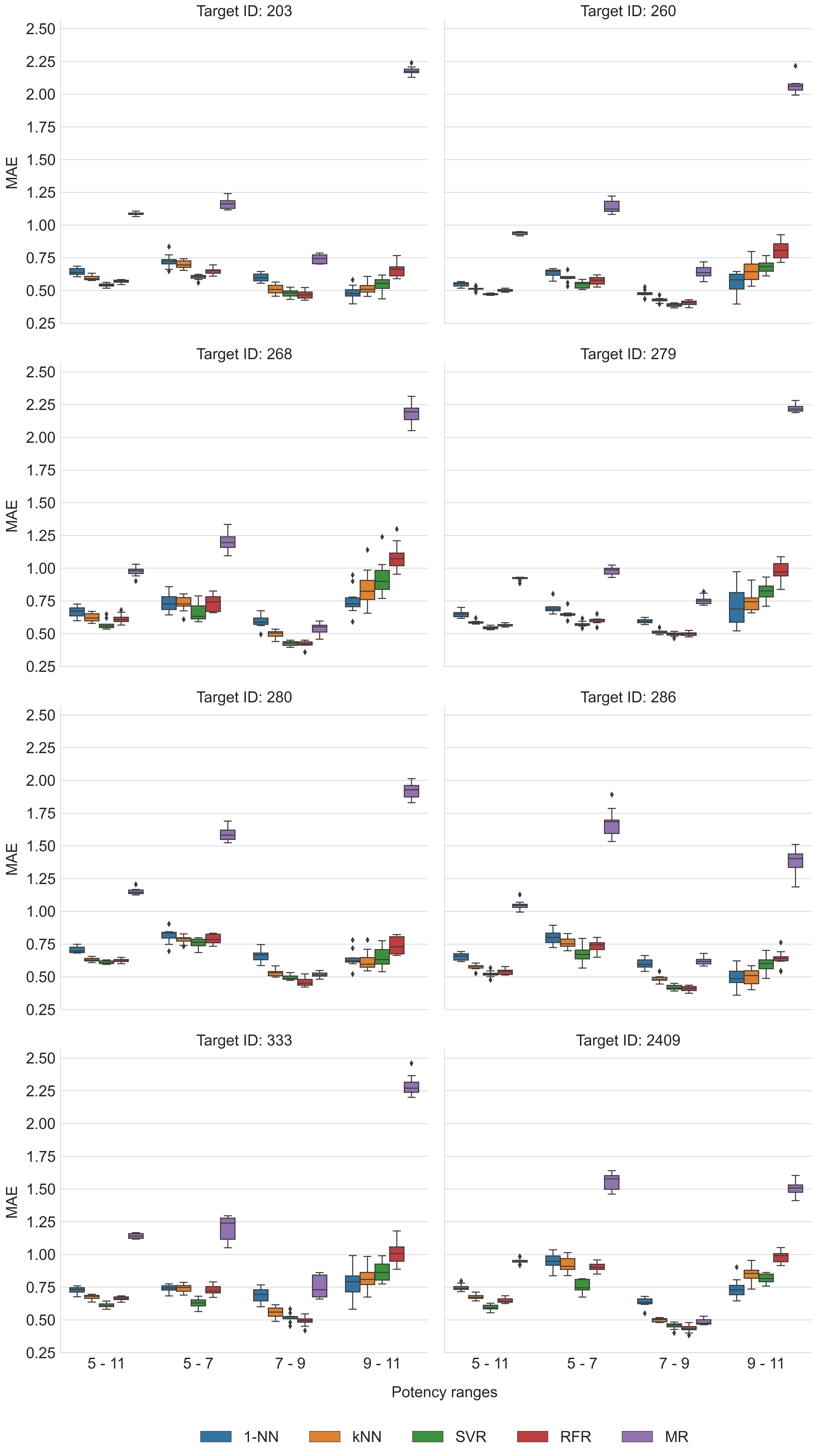


**Fig. S2b**


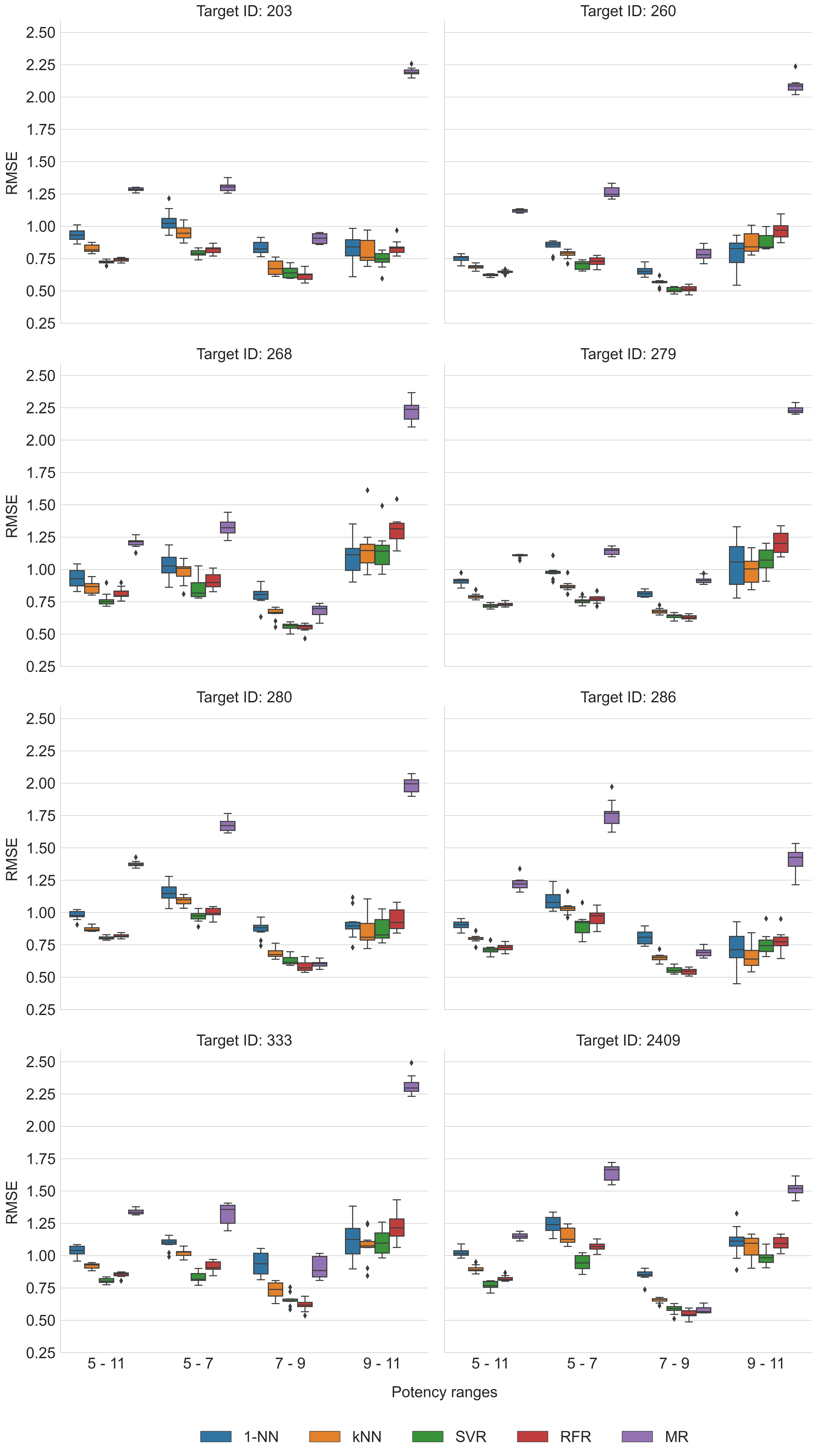


**Fig. S2c**


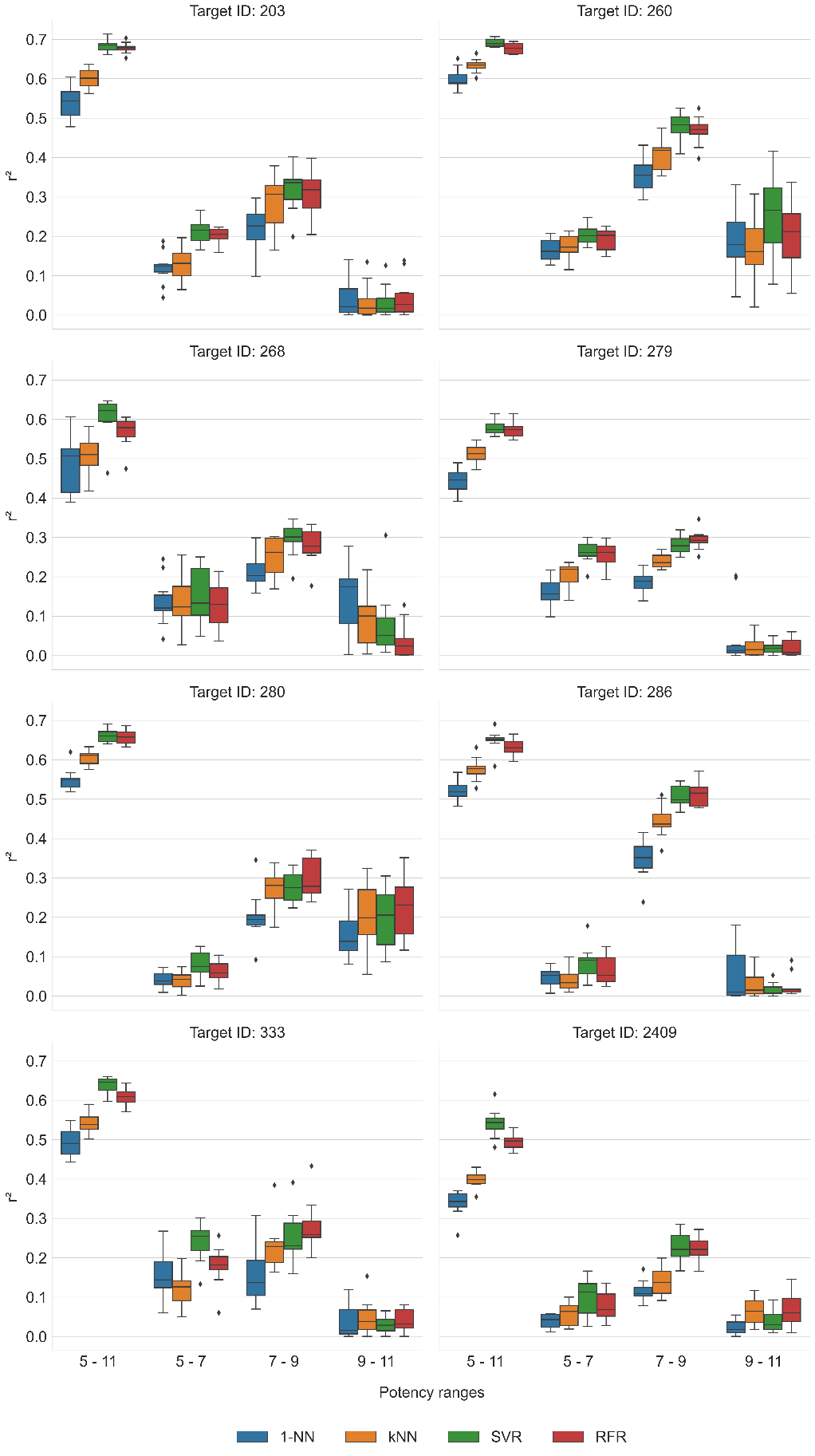


**Fig. S2d**


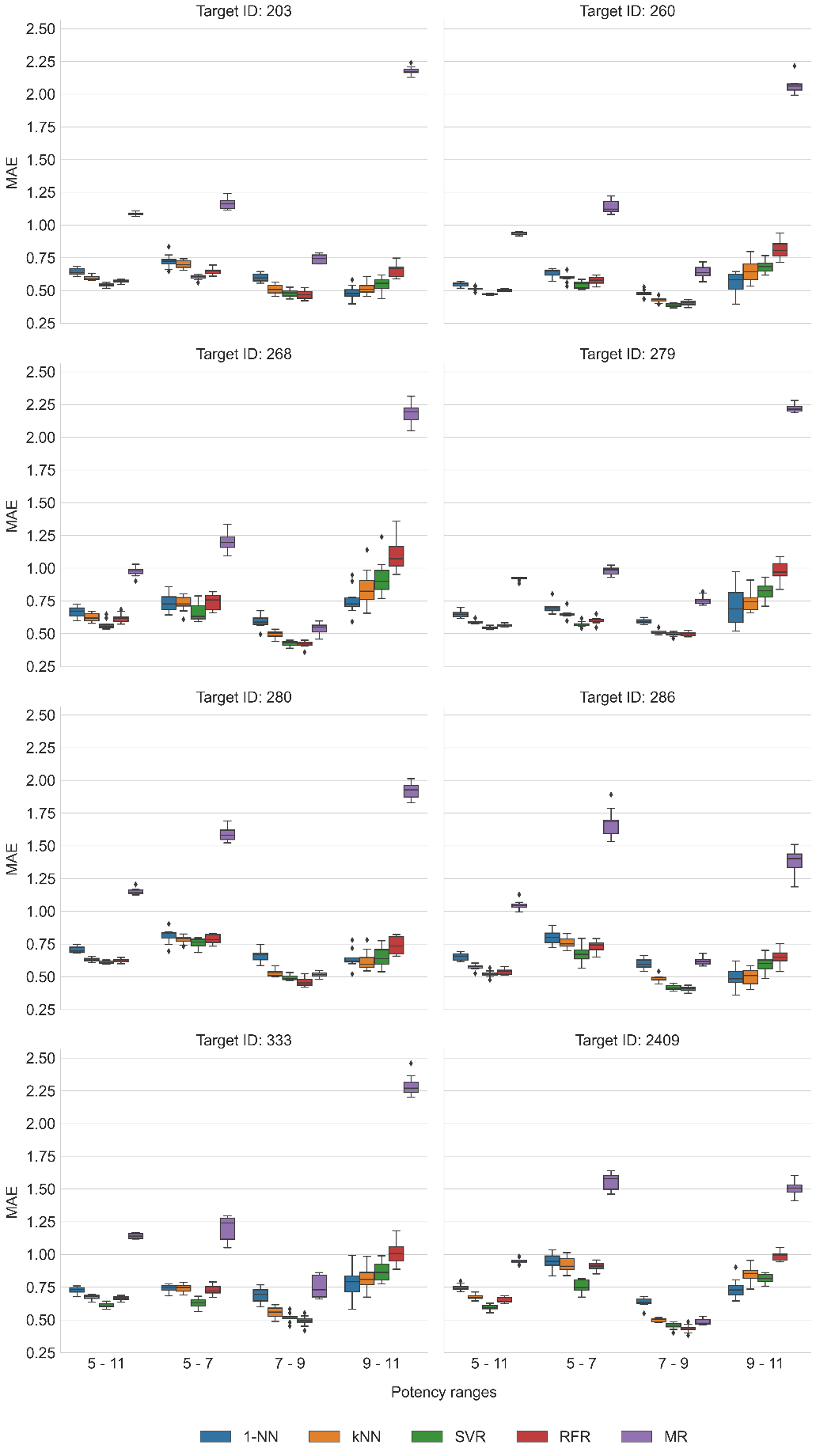


**Fig. S3**


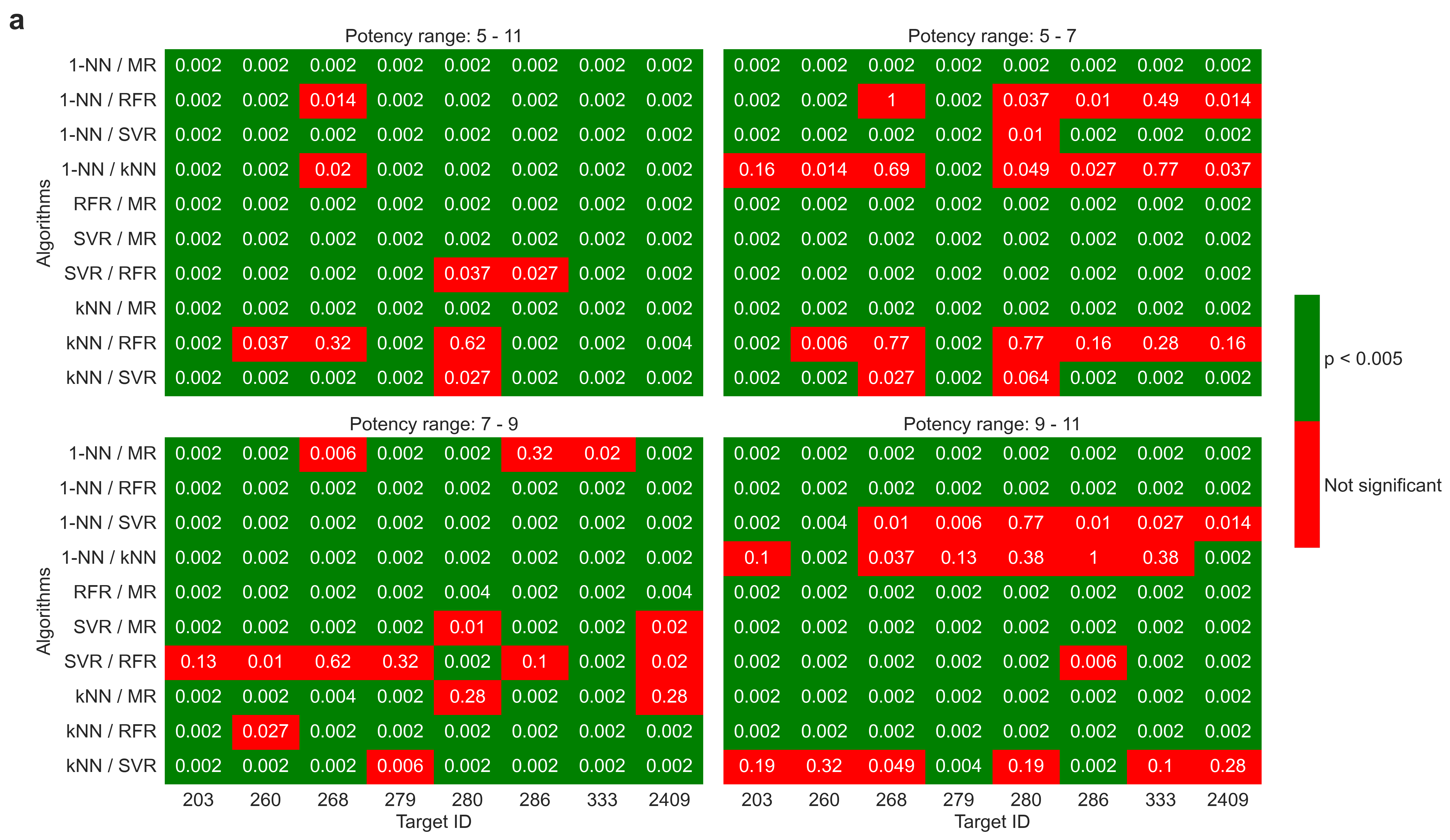


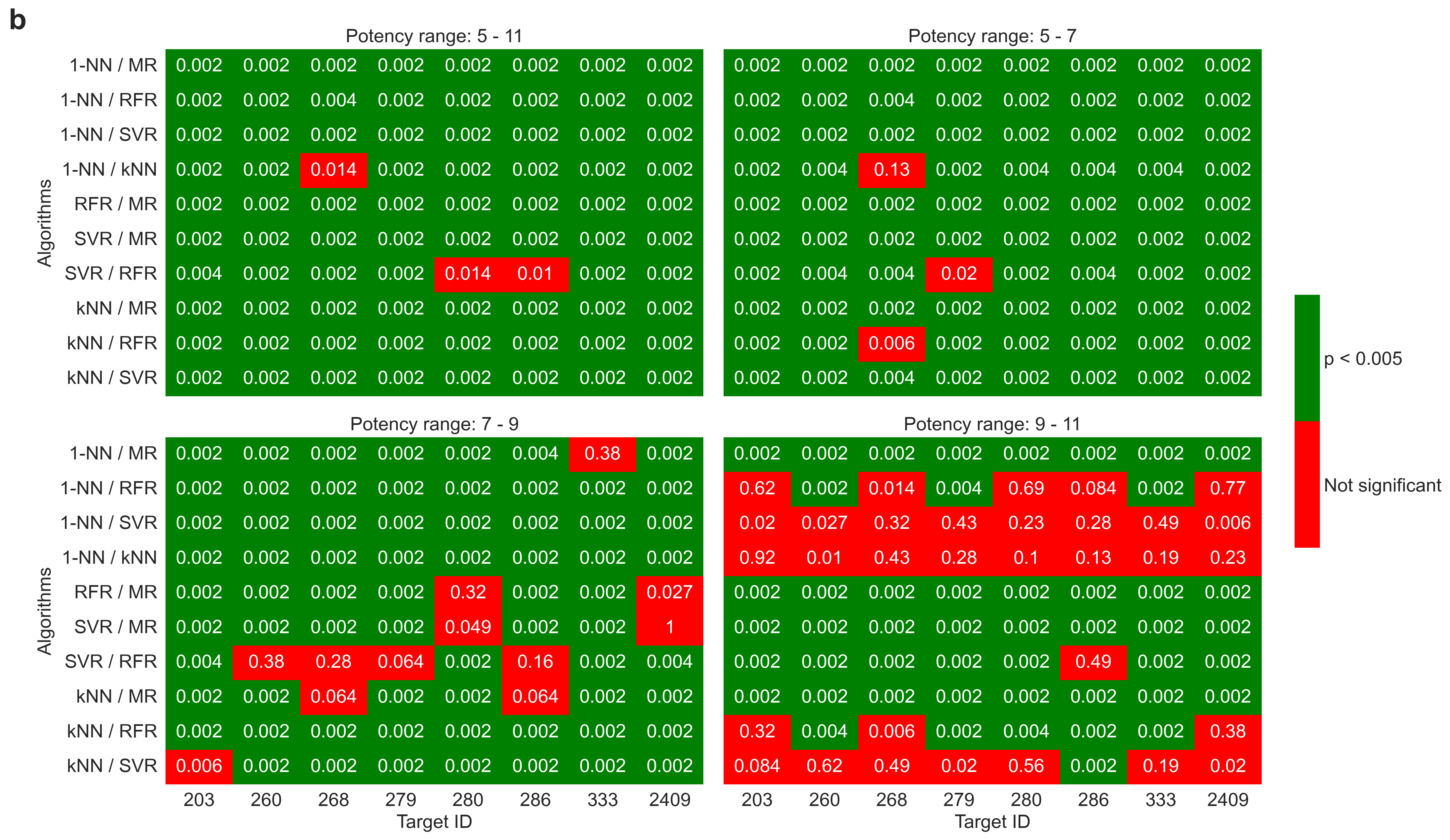


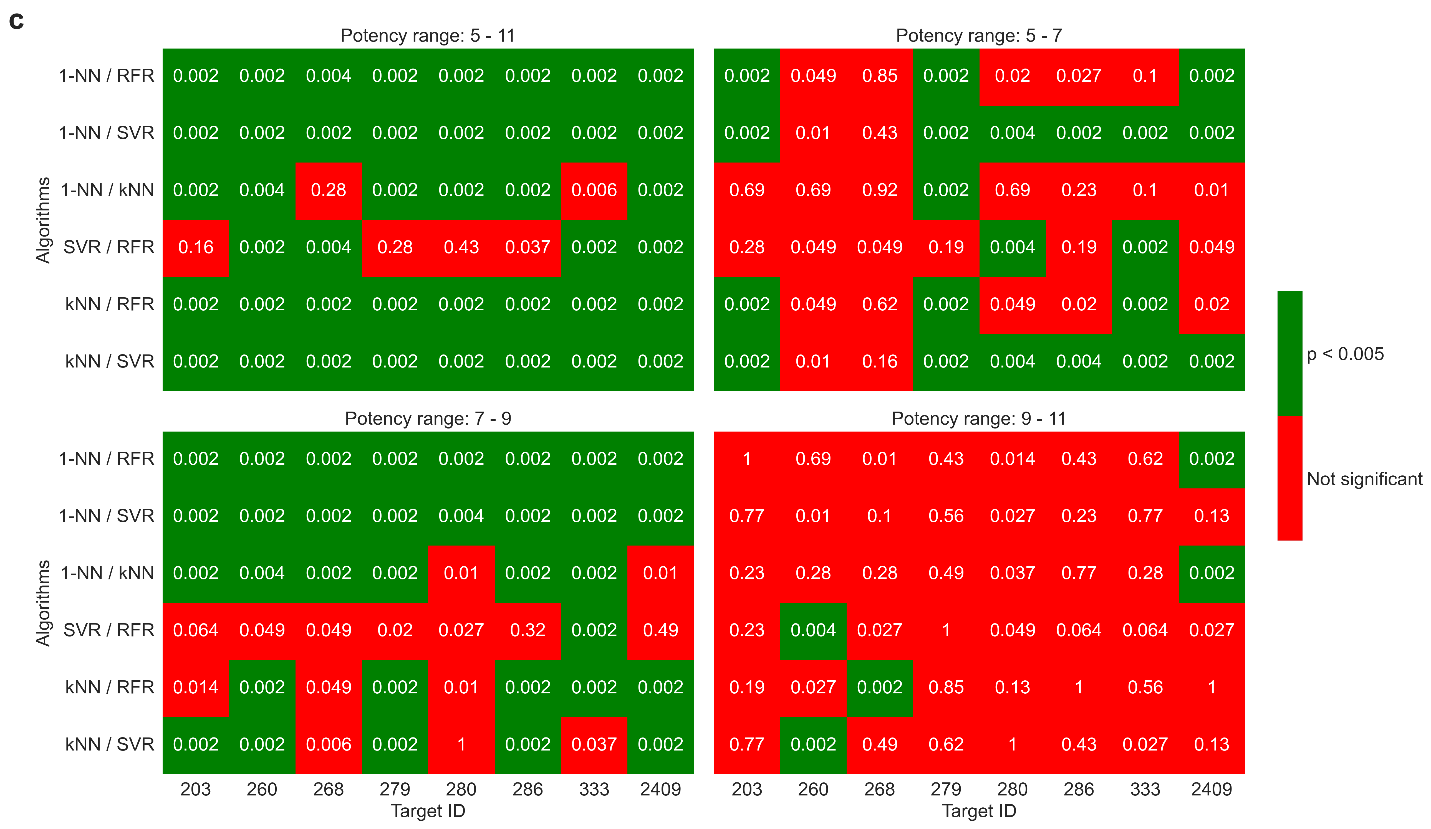


**Fig. S4a**





**Fig. S4b**


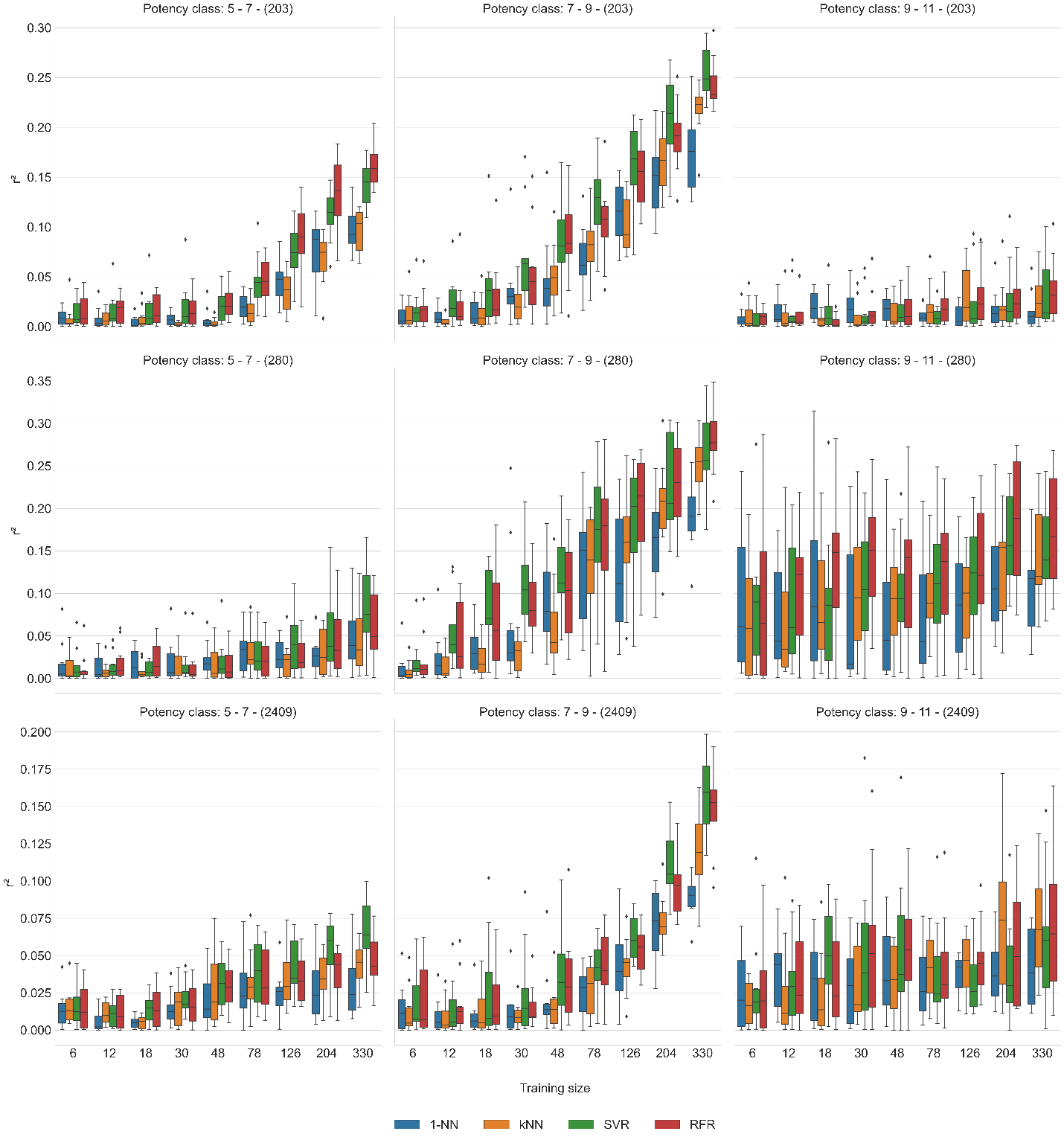


**Supplementary Table 1.** Proportion of compounds per activity class and potency sub-range.

| Target ID | Potency sub-ranges (%) | | |
| --- | --- | --- | --- |
|  | **5 - 7** | **7 - 9** | **9 - 11** |
| 203 | 44.6 | 44.4 | 11.0 |
| 260 | 42.5 | 51.7 | 5.8 |
| 268 | 37.8 | 51.2 | 11.0 |
| 279 | 49.2 | 47.0 | 3.8 |
| 280 | 31.5 | 47.0 | 21.5 |
| 286 | 31.8 | 56.2 | 12.0 |
| 333 | 45.6 | 42.8 | 11.6 |
| 2409 | 31.5 | 56.9 | 11.6 |

Reported are the proportion (%) of compounds from each activity class falling into different potency sub-ranges.
